# The contribution of cardiomyocyte hypercontracture to the burden of acute myocardial infarction: an update

**DOI:** 10.1007/s00395-025-01120-1

**Published:** 2025-06-07

**Authors:** Nur Liyana Mohammed Yusof, Derek M. Yellon, Sean M. Davidson

**Affiliations:** 1 The Hatter Cardiovascular Institute, University College London, 67 Chenies Mews, London, WC1E 6HX UK; 2 Department of Pharmacology, Faculty of Medicine, Universiti Kebangsaan Malaysia, Jalan Yaacob Latif, Bandar Tun Razak, 56000 Cheras, Kuala Lumpur, Malaysia

**Keywords:** Calcium overload, Contraction band necrosis, Hypercontracture, Ischaemia-reperfusion injury, Myocardial infarction

## Abstract

Although reperfusion therapy such as percutaneous coronary intervention and thrombolysis have been implemented in clinical practise as treatments for acute myocardial infarction (AMI) since the 1970s, patients continue to experience high rates of morbidity and mortality. Coronary reperfusion is effective as it limits infarction. However, it induces significant myocardial injury, known as ischaemia-reperfusion (IR) injury. Sustained depletion of cellular adenosine triphosphate (ATP) leading to intracellular calcium (Ca^2+^) overload ultimately lead to cardiomyocyte death during ischaemia. Reperfusion enables resynthesis of ATP, but if this occurs whilst Ca^2+^ remains elevated, it induces excessive cardiomyocyte contracture, known as hypercontracture. Irreversible myocardial injury caused by hypercontracture is often accompanied by histological findings such as wavy myocardial fibres, and more profoundly, contraction band necrosis, identified by the presence of dense eosinophilic bands within the cardiomyocytes. The presence of hypercontracture imposes deleterious effects on both cardiac function and clinical outcomes in individuals experiencing AMI. The potential cardioprotective benefits of inhibiting hypercontracture following IR injury have been demonstrated in animal models, however therapies suitable for clinical application are yet to be developed. This article reviews the pathogenesis and clinical manifestation of hypercontracture in cardiomyocytes during AMI. In addition, the discussion highlights the challenges of translating robust pre-clinical data into successful clinical therapeutic approaches.

## Introduction

According to the Global Burden Disease 2019 Study, ischaemic heart disease continues to make a significant contribution to mortality globally [152], accounting for approximately 40% of fatalities in both men and women in the European Union [121, 176]. A considerable number of deaths arise during acute ischaemic events, namely ST-elevation myocardial infarction (STEMI). However, the mortality rates following STEMI have consistently declined due to advancements in cardiology interventions and the implementation of percutaneous coronary intervention (PCI) [175]. During ischaemia, sustained depletion of cellular adenosine triphosphate (ATP) leads to intracellular calcium (Ca^2+^) overload and ultimately cardiomyocyte death. Early reperfusion (restoration of normal coronary flow) is the optimal therapy for treating those suffering from an acute STEMI as it limits ongoing myocardial infarction (MI). Timely reperfusion therefore remains crucial for salvaging heart tissue [112]. However, return of blood flow paradoxically induces further damage to the heart muscle, termed “lethal reperfusion injury” [59]. This results in cell death and consequent enlargement of the infarct size [135] and increases the risk of subsequent development of heart failure [73].

Reperfusion injury in the myocardium is multifactorial. With the onset of reperfusion, the return of oxygen reactivates mitochondria, and allows the recovery of ATP. This, in combination with high levels of Ca^2+^ causes excessive cardiomyocyte contraction, called hypercontracture. Hypercontracture is defined as an irreversible decrease in maximal cell length of cardiomyocytes, resulting from lack of ATP recovery following prolonged ischaemia in cardiomyocytes, primarily due to Ca^2+^ overload [135]. The occurrence of hypercontracture within the first few minutes of reperfusion appears to be a key factor contributing to cardiomyocyte necrosis [135]. Whilst the phenomenon of hypercontracture has previously been documented in animal models [158], interest has mostly emerged from its recognition as a key pathological feature associated with reperfusion [6]. For example, a multicentre, prospective, randomised controlled trial evaluating distal microcirculatory protection during PCI in STEMI patients demonstrated that although a distal balloon occlusion and aspiration system effectively removed embolic debris, it failed to improve microvascular flow or overall clinical outcomes [169]. This lack of efficacy may be attributed to the intervention being 'too little, too late' to prevent cardiomyocyte necrosis, a process exacerbated by systemic and local inflammatory mediators, endothelial dysfunction, capillary leakage, and interstitial oedema [6]. These findings underscore the complex and multifactorial nature of reperfusion injury, highlighting the urgent need for more effective strategies to address this critical clinical challenge.

Notably, hypercontracture can also cause compression of neighbouring micro vessels, preventing complete reperfusion [107]. This manifestation is widely recognised as “no reflow” or microvascular obstruction (MVO) [36, 67, 91, 99]. For example, when isolated rat hearts were exposed to global ischaemia for 30 min followed by reperfusion for 5 min, a comprehensive no-reflow region was observed in the sub-endocardium. This was accompanied by a reduction of ~50% in coronary flow to the mid-myocardium. This finding suggests that the occurrence of MVO might be correlated with myocardial contracture compression [159]. Despite MVO being an important component of IR injury and contributing significantly to long-term mortality and heart failure [67, 91], there is no treatment available to specifically target it [66]. Inhibition of hypercontracture may therefore have multiple benefits for the reperfused heart. Thus, understanding the pathophysiology underlying cardiomyocyte hypercontracture may pave the way to identifying novel approaches to mitigate the effects of lethal IR injury. This aim of this review is therefore to discuss the pathophysiology and clinical significance of cardiomyocyte hypercontracture that occurs following acute MI (AMI). In addition, the discussion highlights the challenges in translating robust pre-clinical findings into successful therapeutic approaches for patients undergoing clinical treatment for STEMI, emphasising the impact of hypercontracture on the underlying mechanisms of IR injury.

## Cardiomyocyte hypercontracture evidence in patients

In 1974, Bouchardy and Majno identified a unique histopathological pattern called “contraction band necrosis (CBN)” in the hearts of individuals who had suffered from AMI. This discovery has since proven invaluable for pathologists and clinicians in determining the cause of sudden deaths that might otherwise remain unexplained [13]. Numerous autopsy studies from the 1980s, along with a handful of subsequent case reports, validate the similarity between findings in animals and humans. The application of quantitative analysis to assess myocardial CBN can offer valuable information in events of sudden death where AMI has been predicted but not detected using standard histological staining methods [76]. It was also reported that the presence of wavy myocardial fibres in autopsy heart samples from patients was observed at the sub-endocardium. This observation serves as a clinical sign of AMI when the cause of death is unknown [162]. Wavy fibres are often seen in region of myocardial hypercontracture, although they can also be seen in the first few minutes following reperfusion after a brief period of ischaemia not associated with cell death but presumably due to the contraction and stretching that occurs around a non-beating segment of myocardium [33, 87].

An autopsy study found myocardial injury related to coronary artery bypass graft (CABG) surgery, particularly focussing on the paradoxical occurrence of necrosis in areas of revascularisation. The myocardial injury observed was primarily of two types, (1) CBN: this was the most common type, occurring in 82% of patients with transmural necrosis, which predominantly found in areas supplied by patent bypass grafts, (2) coagulation necrosis: this type was less common and was associated with new graft-related coronary artery occlusions. The appearance of regional CBN, which was particularly noticeable in territories supplied by patent vessels, was also associated with sarcoplasmic contraction bands, distinguished by thin, wavy fibre alterations. 1 patient was also reportedly dead within 48 h after the operation, showing a poor prognosis associated with coronary blood reflow [18]. The presence of CBN, along with its distinct histological features, suggests that reperfusion injury after revascularization is a significant contributor to myocardial damage in CABG patients. The poor prognosis in some cases further emphasises the need to address reperfusion-related injury to improve outcomes in cardiac surgery.

Another study analysing 16 autopsied hearts, found that the percentage of the infarct area exhibiting CBN was markedly higher in patients who underwent successful thrombolysis (20% ± 9%) compared to those with unsuccessful thrombolysis (3% ± 3%) [115]. This suggests that CBN is a common pathological finding following reperfusion therapy in AMI cases. In addition, a necropsy study involving 64 cases indicated that the number of myocardial cells containing CBN was significantly elevated in cases of definite MI compared to non-cardiac deaths [76]. This reinforces the notion that CBN is a prevalent feature in AMI, serving as a potential marker for early myocardial injury. However, the studies highlight that whilst CBN is prevalent, its quantification can vary based on the timing and effectiveness of treatment interventions.

**Fig. 1:**
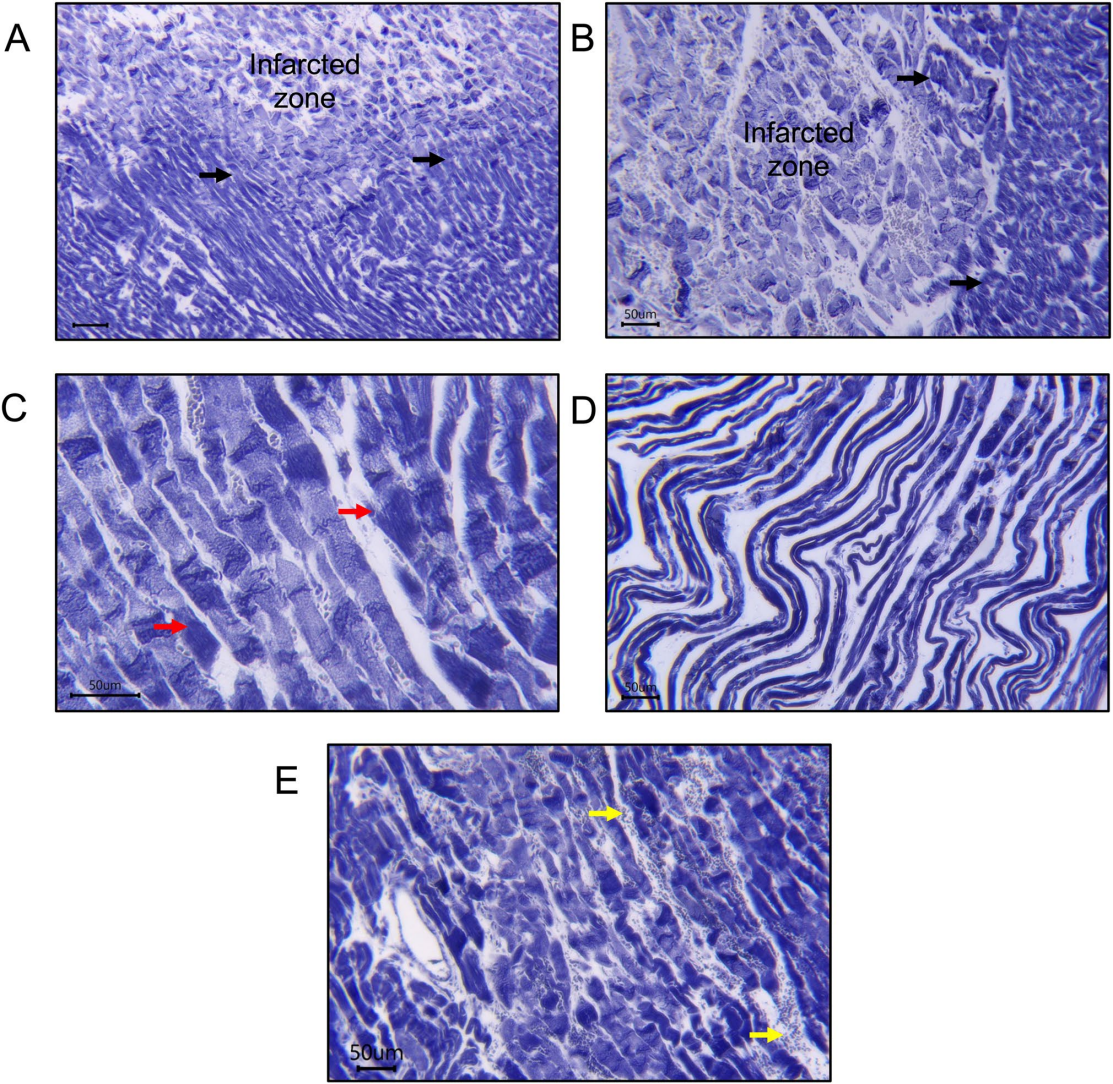
Representative Phosphotungstic acid–haematoxylin staining of transverse sections of rat heart myocardium following 30-min LAD coronary artery occlusion followed by 30 min of reperfusion *in vivo.* **A** The border zone between healthy and infarcted myocardium is clearly differentiated by staining (black arrows). **B** A closer view of the infarcted and border zones (black arrows). The contraction band necrosis (CBN) has propagated extensively throughout the infarcted zone. **C** A closer view of the CBN (dark staining as indicated by red arrows) as a feature of hypercontracture within the infarcted zone. **D** A wavy appearance due to massive myocardium contraction, another common feature of hypercontracture. **E** Extravasation of red blood cells (yellow arrows) in between myocardial fibres, indicating the presence of intramyocardial haemorrhage. Bars = 50 μm

Interestingly, CBN was also accompanied by microvascular injury leading to endothelial swelling, MVO, and intramyocardial haemorrhages (IMH), which were commonly found in AMI patients [190]. Many theories have attempted to elucidate how myocardial reperfusion causes injury to the microvasculature, which is often seen as IMH [33, 68, 183]. IMH occurs as leakage of red blood cells through the injury of endothelial walls [6, 140] leading to the breakdown of blood vessel structure, allowing erythrocytes to escape into the surrounding tissue [75, 131]. Erythrocyte extravasation and neutrophil accumulation are histological observations that may be indicative of IMH and are also seen in animal model of IR (Fig. 1). Recently, cardiac magnetic resonance imaging in a longitudinal clinical study amongst STEMI patients after primary PCI demonstrated the occurrence of IMH [109]. This IMH evolved as a second wave front expansion led to a larger infarct size, and notably, it occurred long after the application of reperfusion therapy. The occurrence of MVO related to the IMH wave front significantly enlarged the extent of the heart tissue damage, largely diminishing the beneficial effects achieved through the earlier reperfusion treatment. In addition, IMH negatively impacts cardiac function. This was demonstrated by increased LV end-diastolic and systolic volumes and lower LV ejection fractions (LVEFs) compared to patients who do not experience IMH. This cardiac function was reported to worsen significantly, with reductions in EF and adverse LV remodelling observed after 7 months of follow-up post-PCI [20], eventually associated with unfavourable clinical outcomes and an increased risk of death [9, 109].

## Cardiomyocyte hypercontracture in animal studies

In 1960, Jennings et al. documented the features of reperfusion injury in the hearts of canines histologically, demonstrating the ultrastructural appearance of “massive cardiomyocyte swelling” with architectural disruption, contraction bands, and intramitochondrial calcium phosphate granules [83]. The ultrastructural changes of rapid cellular swelling or tissue oedema including organelles such as mitochondria and formation of large blebs of fluid beneath the sarcolemma were observed in early reperfusion. Under light microscopy, these blebs appeared as extracellular swelling [189]. Similarly, following just 2 min of reperfusion, severely swollen cells exhibiting contraction bands were observed alongside cells showing ischaemic changes identical to those seen after 40 min of ischaemia without reperfusion. At 5- and 10-min reperfusion, similar morphological features were present, though more contraction band-containing swollen cells were observed [93]. The mechanism of this cellular swelling is believed to result from either Na⁺-K⁺ pump failure or a substantial increase in membrane permeability that exceeds the pump's ability to remove Na⁺ [189]. In addition, metabolic inhibition induces cellular swelling, likely due to inadequate ATP generation to maintain Na⁺-K⁺ pump activity. Consequently, Na⁺, Cl⁻, and water passively enter the cells following the osmotic gradients [94].

In later studies, Piper and colleagues gained significant insight into hypercontracture in IR injuries. Their research predominantly employed a simulated ischaemia model involving anoxia, cell acidosis, and substrate depletion in isolated adult ventricular cardiomyocytes *in vitro*. Using this model, they measured the temporal changes in irreversible cell shortening in a condition of ATP depletion, resulting in a type of contracture, called rigour contracture and following reoxygenation (leading to further cardiomyocyte hypercontracture). Their hypothesis was that the prolongation of contracture during reoxygenation, which contributes to irreversible cell injury, causes Ca^2+^ overload during ATP recovery (referred to as the Ca^2+^ paradox) [137, 157].

Histologically, hypercontracted cells are often identified in regions defined as CBN in haematoxylin and eosin staining myocardial sections. An alternative histological method involves phosphotungstic acid haematoxylin (PTAH)-staining [78, 148, 167] (Fig. 1). Serial histological observations following the return of ATP to the ischaemic cardiomyocytes suggest that sarcolemma damage and cell death occur as a consequence of the high forces of mechanical stretch generated by hypercontracted cardiomyocytes [62, 83]. In the 1980s, Jennings and colleagues showed that irreversible cardiomyocyte injury in canine models of IR injury was observed with several features, which included a markedly swollen sarcolemma and CBN. Upon ultrastructural observation, severely damaged mitochondria with a prominent irregular shape of cristae and matrix space were even more prominent [85, 86, 93].

The occurrence of hypercontracture was also associated with the events of arrythmias [129, 196]. Reversible arrhythmias typically occur shortly after a brief episode of ischaemia and rapidly upon reperfusion. These arrhythmias manifest as rhythm disturbances during partial or complete restoration of blood flow in previously ischaemic tissues. Parker et al. identified ventricular arrhythmias in rabbit hearts *subject* to IR *ex vivo* [129]. The contribution of altered intracellular Ca^2+^ handling and subsequent arrhythmias during acute ischaemia is of special interest, as it is also playing a crucial role in cardiomyocyte hypercontracture. During reperfusion, the mechanical stresses experienced can influence the electrical properties of heart cells. Hypercontracted cells resulting from changes in ion channel function during reperfusion can create a substrate that is conducive to the development of transient arrhythmias. It is hypothesised that these modifications play a role in the electrical uncoupling that induces ventricular arrhythmia in the context of myocardial ischaemia [118]. Similarly, Tribulova et al. demonstrated subcellular myocardial alterations when hearts were subjected to various acute proarrhythmogenic conditions [178]. Using an intracellular calcium imbalance model in isolated hearts to induce proarrhythmogenic conditions, whilst promoting a sudden increase in catecholamines and heart rate, they found that a common feature of ultrastructural alterations such as hypercontracted cardiomyocytes and contraction bands of myofibrils preceded the occurrence of life-threatening cardiac arrhythmias in rats, guinea pigs, and Landrace pigs. In short, the findings highlight how disruptions in intracellular calcium levels and myocardial cell-to-cell connexions play a significant role in the development of arrhythmias, affecting the ultrastructure of cardiomyocytes and their junctions [148]. Although the occurrence of arrhythmias is generally infrequent, it is crucial to manage them promptly to prevent potential complications, including those that are life-threatening [96, 111].

## Ischaemia-induced contracture

The acute effects of ischaemia on myocardial tissue were first described by Jennings in the 1960s [84]. Acute ischaemia frequently results from a blockage in the circulation of the coronary artery, primarily due to thrombosis or the breakdown of atherosclerotic plaques [17]. The deficiencies of oxygen and nutrient supply in the coronary circulation lead to a series of structural, functional, metabolic, and biochemical alterations in the heart. The cardiomyocytes become vulnerable, which increases plasma membrane permeability, and the cell eventually undergoes necrosis. This injury to the cardiomyocyte is associated with reductions in cardiac cell density, structural integrity, and perfusion capacity [17, 59, 84].

In the absence of oxygen, oxidative phosphorylation by mitochondria immediately ceases, leading to loss of mitochondrial ATP production, mitochondrial membrane depolarization, and decrease in reserve myocardial contractile function [16, 17, 59]. Lack of oxygen results in a shift towards anaerobic glycolysis for ATP production. This reaction pathway reduces pyruvate to lactate. Cells can experience changes in pH (< 7.0) due to altered metabolism and accumulation of hydrogen ions (H^+^). During ischaemia, the acidic intracellular conditions cause the mitochondrial permeability transition pore (MPTP) to remain close. The accumulation of intracellular H^+^ causes the sodium–hydrogen exchanger (NHE) to facilitate its release from the cell in exchange for the entry of sodium (Na^+^), leading to an elevated intracellular Na^+^ concentration . In addition, the Na^+^–K^+^ ATPase stops functioning as a consequence of ATP depletion, further exacerbating Na^+^ overload inside the cells [63, 143]. Hence, the role of the NHE in ischaemia was previously studied. In early study using a pig model of IR, with 36 min of ischaemia, the NHE inhibitor, cariporide delayed the onset of contracture and reduced ATP depletion [50]. Using an *ex vivo* model of IR, isolated hearts from the wild-type (WT) mice developed ischaemic contracture (as indicated by a rise in LV end-diastolic pressure, LVEDP) after 20 min of ischaemia, exhibit maximal contracture at 30 min of ischaemia. The NHE1^−^/^−^ mice, on the other hand, showed a significant reduction in myocardial contracture (LVEDP of 54.2 ± 3.7 mmHg in NHE1^−^/^−^ hearts vs 83.6 ± 5.7 mm Hg in WT hearts) [187]. Histologically, in contrast to NHE1^−^/^−^ hearts, which have a majority of cells that are nearly normal with visible nuclei and just a small number of cells that have undergone hypercontracted (contraction band) myofibers, WT hearts subjected to IR injury exhibit a visible structure damage with hypercontracted myofibers. These findings support the idea that inhibition of Na^+^ overload via the NHE may be cardioprotective [187].

As the sarcoendoplasmic reticulum (SR) Ca^2+^ ATPase (SERCA) pump relies on ATP to function, prolonged ischaemia causes an impairment in the SR Ca^2+^ uptake [108], thereby serving as triggers for the intracellular influx of Ca^2+^. In addition, under normal conditions, the primary function of the Na^+^–Ca^2+^ exchanger (NCX) is to release Ca^2+^ out of the cytosol in exchange for Na^+^ in the cells. Nevertheless, during ischaemia, a depolarized plasma membrane and intracellular Na^+^ overload results in Ca^2+^ influx via NCX acting in reverse mode, which results in further excess cytosolic Ca^2+^. The mitochondrial Ca^2+^ uniporter subsequently transports cytosolic Ca^2+^ into the mitochondria [41], causing an increase in the activation of Ca^2+^-sensitive mitochondrial matrix enzymes [170]. The described mechanisms are primarily involved in the development of ischaemic contracture, leading to the generation of rigour force and stiffness in cardiomyocytes, with an overload of Ca^2+^ as the main mediator [97, 180].

The development of ischaemia-induced cardiomyocyte injury is facilitated by extensive membrane damage resulting from multiple mechanisms. For instance, the accumulation of cytosolic ions results in marked osmotic swelling [39]. This eventually disrupts the sarcolemma and activates necrotic cell death cascades [89]. The ultrastructural changes of the ischaemic cells with metabolic disturbances include SR swelling and breakdown of the mitochondrial membrane. Structural alterations also occur in the microvasculature and myocardial interstitial as the cell injury progresses.

Faber et al 1981 extensively discussed the concept of reversible and irreversible cell injury during ischaemia. With shorter period of ischaemia, myocardial mechanical function, membrane potential, metabolism, and ultrastructure can recover from ischaemic injury. However, prolonged ischaemia leads to irreversible damage in the affected cells. Even with reperfusion, these cells continue to deteriorate and undergo necrosis. Extended ischaemic periods will further result in biochemical changes that differentiate irreversibly injured cells from those that are reversibly damaged [35]. When the left coronary artery was occluded for 40 minutes in dogs, the affected myocardial cells became 'irreversibly injured’ and progressed to necrosis and were ultimately replaced by scar tissue [15, 21]. Ischaemic durations of longer than 20 min may produce extensive tissue injury and eventually lead to cardiomyocyte death if not prevented [17, 145, 192]. Prolonged ischaemia (up to several hours) also causes cellular necrosis, which leads to a subsequent inflammatory response [30]. Further, the cardiomyocyte swelling and/or hypercontracture can cause mechanical compression of the microvessels. The compression of microvessels within the myocardium indicates the potential impairment of coronary flow into the ischaemic region, which may result from damage to the microvessels, or compression caused by the swollen myocytes, leading to MVO [93, 95].

## Reperfusion-induced contracture

During reperfusion, the sudden reintroduction of oxygen into previously oxygen-deprived heart tissue causes further damage, which is referred to as the “oxygen paradox” [125]. It is plausible that some cells, already on the verge of irreversible damage at the end of ischaemia, experienced exacerbated injury during reperfusion. This secondary insult may be driven by mechanisms such as calcium overload [158], oxidative stress [28], and mitochondrial dysfunction [30], which collectively push these critically compromised cells towards cell death. Despite its potential to salvage ischaemic myocardium, reperfusion itself can induce several forms of histologically observable damage, including hypercontracture and sarcolemma disruptions. The “stone heart” phenomenon arises upon reperfusion of hearts following extended periods of ischaemia. It is characterised by stiffness and a pale heart as a result of substantial hypercontracted muscles and ruptured cellular membrane leading to the subsequent loss of intracellular content into the extracellular space such as nucleic acid & mitochondrial DNA, inflammatory markers, and even danger-associated molecular patterns (DAMPs) that acts as damage signals to the cells [8, 25]. This stone heart phenomenon typically takes place during ischaemia and worsens during the initial phases of reperfusion [135, 139]. Ultrastructural changes in the stone heart include SR and T-tubule distension. Myofibrillar degeneration is also prominent [106]. Another important key feature of hypercontracture and myocardial necrosis following IR is CBN [38]. Histologically, CBN reflects the presence of hypercontracted myofibers and sarcolemma disruption and appears as dense, eosinophilic bands within the affected muscle fibres, as shown in Fig. 1. It is attributed to the presence of powerful and homogeneous mechanical forces exerted by cardiomyocytes in the presence of ATP and high cellular Ca^2+^ [182]. Another distinctive feature of hypercontracture following AMI is the pronounced wavy appearance of myocardium resulting from the extensive muscle contraction. A study using BDM's inhibition of segmental wall shortening and decrease in end-diastolic length during coronary occlusion and reperfusion reduced infarct size and significantly decreasing CBN. These findings emphasise the importance of understanding reperfusion-induced hypercontracture as a potential target to reduce IR injury [42].

There are two commonly described mechanisms of reperfusion-induced hypercontracture, namely: Ca^2+^ overload contracture, and rigour-type contracture [136]. Ca^2+^ overload-induced contracture occurs when cardiomyocytes are rapid re-energised following episodes of Ca^2+^ accumulation during ischaemia. Uncontrolled mechanical stretch of cardiomyocytes is mainly caused by high cytosolic Ca^2+^ in the presence of ATP [2, 135, 136]. However, if cardiomyocyte reoxygenation occurs at a very slow pace, rigour-type contracture may be induced after a prolonged period of ischaemia [5, 12]. These two distinct causative pathways for reperfusion-induced hypercontracture are further discussed in the following sections.

## Involvement of calcium overload-induced contracture

**Fig. 2:**
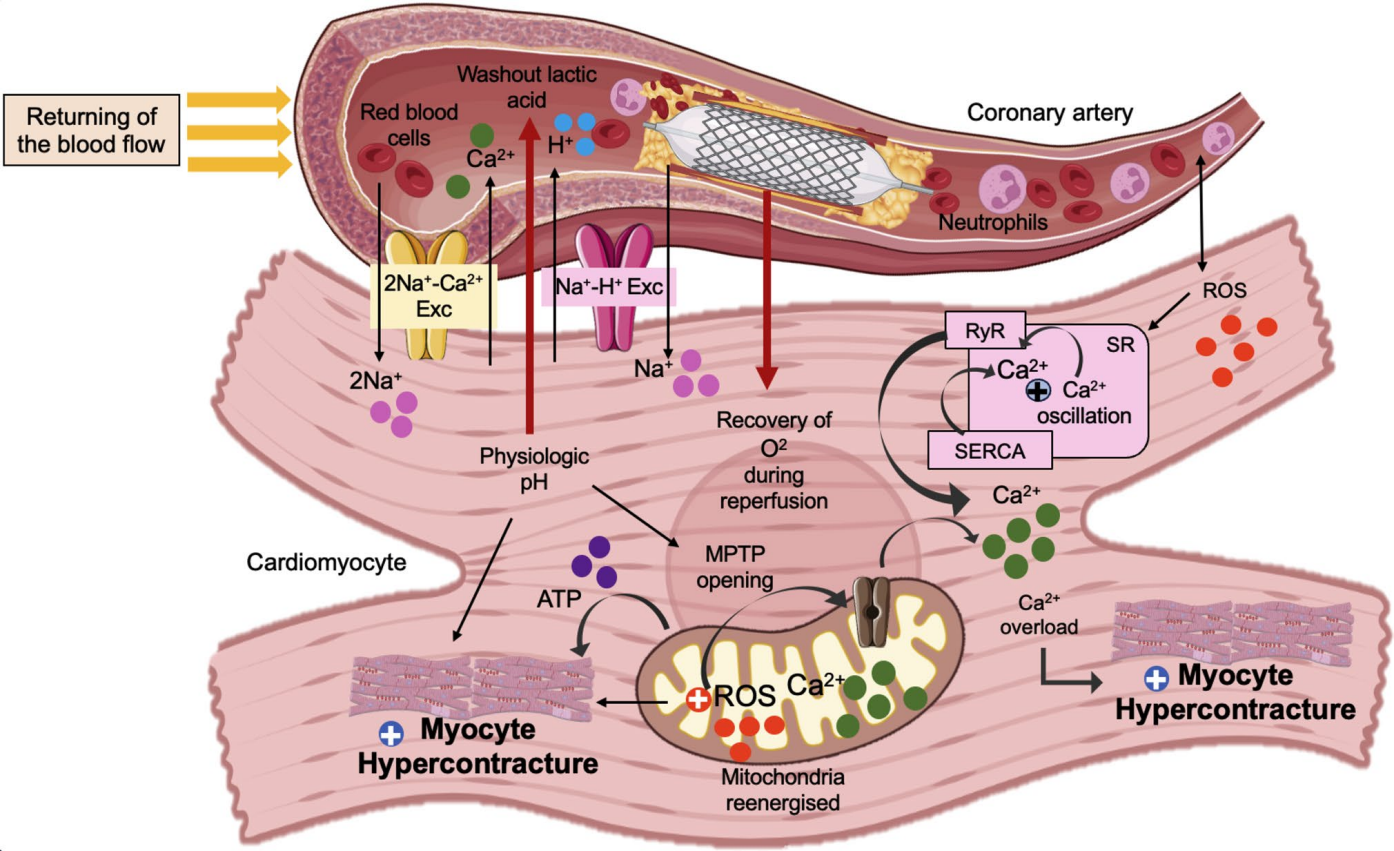
The main mediators of cardiomyocyte hypercontracture during myocardial reperfusion. During acute myocardial infarction (AMI), no blood supply causes shifting cellular metabolism towards anaerobic glycolysis, promoting in the generation of lactate, the accumulation of protons, and a change in intracellular pH. The mitochondrial permeability transition pore (MPTP) remains close during this event. As a consequence, intracellular sodium (Na^+^) and calcium (Ca^2+^) levels rise. Returning of the blood flow to ischaemic myocardium, however, results in various physiological alterations within the cardiomyocytes. Subsequent Ca^2+^ influx, released by the sarcoplasmic reticulum (SR) in response to the existing intracellular Ca^2+^ overload, alongside ATP recovery, promotes the reopening of the mitochondrial permeability transition pore (MPTP). Rapidly normalising physiological pH and the production of ROS consequently induce cardiomyocyte hypercontracture. (Images from BioRender: https://BioRender)

Ca^2^⁺ overload is exacerbated when oxygen is rapidly reintroduced during reperfusion, leading to simultaneous energy recovery and elevated intracellular Ca^2^⁺ levels, a phenomenon known as the Ca^2^⁺-overload mechanism [158]. If the mitochondria remain structurally intact during ischaemia, reoxygenation promptly restores oxidative phosphorylation [28]. Resynthesis of ATP promotes cardiomyocytes’ recovery from cytosolic cation imbalance whilst also reactivating actin-myosin interactions. However, this reactivation of actin-myosin cross-bridging is usually more rapid than the recovery of cellular Ca^2+^ imbalance, resulting in an uncontrolled Ca^2+^ hypercontracture [155, 161]. The pathophysiological mechanisms that lead to hypercontracture during myocardial IR injury are illustrated in Fig. 2.

As cellular acidification produced by anaerobic respiration induces Na^+^ overload through the NHE, which increases cardiomyocyte membrane Na^+^ permeability, the Na^+^/K^+^-ATPase activity is further decreased [5]. Subsequently, the SR cyclically uptake and releases Ca^2+^, which enhances excessive Ca^2+^ overload-induced hypercontracture [135]. Upon reperfusion, the transarcolemmal Na^+^ gradient continues to be lowered, and the NCX remains active in reverse mode [5]. Ischaemia causes myofibrils to contract less effectively because of low intracellular pH, even if cytoplasmic Ca^2+^ concentrations are high throughout the period of ischaemia [49]. Nevertheless, during reperfusion, elevated ATP levels coupled with Ca^2+^ overload and pH restoration triggers massive myofibril contraction, eventually provoking cell death due to sarcolemma rupture [148].

Ca^2+^ overload-induced hypercontracture is also associated with the opening of the MPTP. During reperfusion, high cytosolic calcium content and resumption of ATP cause MPTP opening [27]. Ruiz-Meana et al. demonstrated that when Ca^2+^ overload was stimulated in isolated, permeabilized cardiac myocytes in the presence of ATP, MPTP opening can provoke hypercontracture, and was inhibited when ATP levels were reduced [153]. The opening of the MPTP allows Ca^2+^ to move freely across the inner mitochondrial membrane, and leads to mitochondrial swelling [27, 77]. This swelling can disrupt the structural integrity of the mitochondria and compromise their normal function. High levels of mitochondrial Ca^2+^ further disrupt various processes, including the electron transport chain (ETC) complexes and ATP synthesis, causing cell dysfunction and injury [77]. Subsequently, rupture of the mitochondrial inner membrane leads to the release of cytochrome C from the mitochondria into the cytosol. Cytochrome C is a key component in the ETC complexes, and its release triggers the activation of apoptotic pathways, ultimately leading to programmed cell death (apoptosis) [71, 98, 134].

## Role of reoxygenation-induced rigour contracture

In comparison to contracture caused by Ca^2+^ overload, rigour-type contracture causes less pronounced cell shortening. Cardiomyocytes typically retain their rod-shaped structure. This takes place under low ATP conditions and is not initiated by cytosolic calcium overload [60, 120, 154]. The initiation of reoxygenation is linked to the development of this substantial hypercontracture-induced myocardial damage [38, 61]. The term ‘oxygen paradox’ (as mentioned in section “Reperfusion-induced contracture” above) has been used to refer to this event in which re-energisation promptly induces severe cardiomyocyte damage. As ischaemia-related cellular injury progresses, the rapid restoration of cellular ATP by mitochondria diminishes upon reoxygenation [27]. Opening of the MPTP disrupts mitochondrial oxidative phosphorylation, eliciting a fall in the mitochondrial membrane potential and impaired mitochondria function. This event ultimately results in cellular death through necrosis [53]. At the subcellular level, studies have demonstrated that a rigour contracture occurs when intracellular ATP concentrations fall within the sub-millimolar range (less than 100 mol/l) [3]. Within cardiomyocytes, slow turnover of cross bridges may be caused by very low ATP, hence inducing rigour contracture [101, 122]. This type of contracture may be mediated via Ca^2+^-independent stimulation of actin-myosin crossbridge formations that have already developed rigour bonds (during ischaemia) at several parts of their crossbridge sites. In rigour contracture, if ATP is not available or is depleted, the myosin heads remain strongly bound to actin, leading to sustained contraction and the formation of rigour bonds [101, 138]. In contrast to ischaemia, when cardiomyocytes undergo reoxygenation after a period of low oxygen, they often remain in a prolonged state of low ATP availability to allow the gradual recovery of oxidative phosphorylation and energy production. This extended ATP depletion during reoxygenation can exacerbate hypercontracture severity, leading to excessive cell shortening [138]. Table 1 below further summarises the comparison of the phenotypic and mechanistic distinctions between Ca^2^⁺ overload-induced contracture and IR-induced rigour-type contracture.

**Table 1:** Comparison of phenotypic and mechanistic distinctions between Ca^2^⁺ overload-induced contracture IR-induced rigour-type contracture

Feature	Ca^2+^ overload-induced contracture	IR-induced rigour contracture	References
Trigger	Calcium overload during prolonged ischaemia with fast recovery of ATP during reperfusion	ATP depletion in brief period of ischaemia, may also occur during ATP reintroduction to the ischaemic cardiomyocytes at a very low rate and slow recovery of ATP	[38, 139]
ATP levels	Present initially but slowly decline depending on the duration of ischaemia	Severely depleted (< 100 μmol/L)	[3, 122]
Calcium involvement	Elevated cytosolic calcium initiates hypercontracture due to reverse mode of NCX	Not essentially depends on calcium levels, but presence of low ATP during reoxygenation provoke contracture	[139]
Morphology	Super contracted sarcomeres (shortening), contraction bands, ruptured membranes upon reperfusion	Intact sarcomeres, condensed cytoplasm, intact membranes early	[3, 156]
Reversibility	Irreversible if sustained	Potentially reversible with rapid ATP reintroduction, irreversible if prolonged	[38, 70]
Associated with	Reperfusion injury, CBN	Ischaemic core zones	[70]

Summary of phenotypic and mechanistic distinctions between Ca^2^⁺ overload-induced contracture and IR-induced rigour-type contracture, highlighting key differences in their underlying mechanisms and physiological manifestations. NCX: sodium–calcium exchanger

## The spread of myocardial hypercontracture

**Fig. 3.**
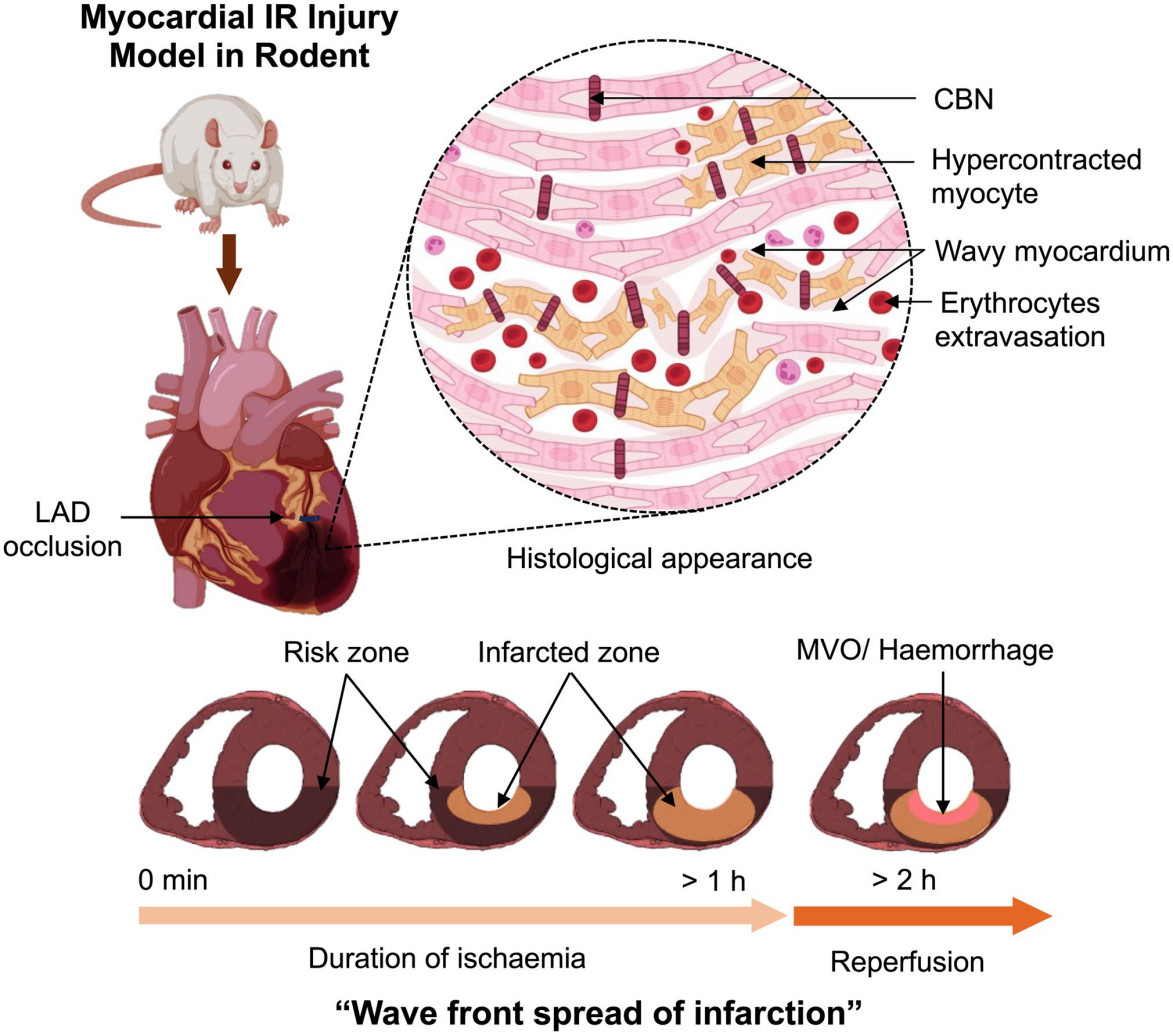
The wavefront spread of myocardial infarction in an ischaemia-reperfusion (IR) injury model. As the duration of ischaemia increases, the infarcted zone expands, possibly covering a larger portion of the risk zone. Histologically, the ischaemic myocardium exhibits a wavy appearance, hypercontracted cardiomyocytes (also known as contraction band necrosis, CBN), and intramyocardial haemorrhage, as indicated by erythrocyte extravasation, which eventually progresses to the no-reflow phenomenon or microvascular obstruction (MVO). (Images from BioRender: https://BioRender)

The concept of a “wavefront” in MI by Reimer & Jennings, was developed to explain how the ischaemic process causing myocardial necrosis evolves over time, originating from the endocardial regions and progressing towards the epicardium [146] (Fig. 3). The duration of myocardial ischaemia significantly affects the severity of myocardial necrosis. Short periods of myocardial ischaemia do not lead to substantial injury, making it challenging to detect a measurable enhancement in myocardial salvage. Nevertheless, prolonged periods of myocardial ischaemia not only lead to IR injury but can also influence the effectiveness of the cardioprotective approach employed [19, 47].

Whether myocardial hypercontracture and CBN also progress through the reperfused myocardium in a wavefront is unknown [42]. However, during AMI, the propagation of myocardial necrosis originates at the sub-endocardium and extends outward in a wavefront across the epicardium [147]. Unsurprisingly, the regional distribution pattern of myocardial hypercontracture corresponds to myocardial necrosis [165]. In longitudinal sections of heart tissue, hypercontracture of the myocardium is evident within the infarct zone and progressively diminishes towards the peripheral boundaries, commonly referred to as the border zone [22, 130]. Specialised staining techniques, such as PTAH, allow for the differentiation between the infarcted zone and the border zones (Fig. 1). CBN is unique due to its hypercontraction bands, calcium-mediated damage, and association with reperfusion injury, unlike other forms of cell death such as necrosis or apoptosis. Accurately determining how much each cell death mechanism contributes to total infarct size during MI is difficult [69, 80]. Commonly used techniques for measuring infarct size using triphenyl tetrazolium chloride staining or gadolinium-based imaging cannot differentiate between different modes of cell death [12]. The role of each cell death pathway may differ between different cell types [69]. For instance, apoptosis is more prominent in non-cardiomyocytes compared to cardiomyocytes [30]. Hence, Table 2 summarises the differences between CBN and other type of cell death mechanism, highlighting key components and mediators for each type.

**Table 2:** Key differences between CBN and other type of cell death mechanism and its contribution to infarct size

Type of cell death	Trigger/initiation	Key mechanisms	Morphology	Sarcolemma integrity	Inflammatory response	Role in IR injury/infarct size
Necrosis	Occurrs during prolonged ischaemia (> 20–40 min), mainly due to ATP depletion [69]	During ischaemia: Failure of Na⁺/K⁺-ATPase → Na+ overload → Ca^2^⁺ overload (due to reverse mode of NCX) [102, 174] Upon reperfusion → ROS production → proteins, lipids, and DNA damages [70, 141]	Cell swelling, mitochondrial and sarcolemmal rupture [146, 147] , contraction bands → membrane rupture and cell death, loss of nuclei [139]	Impaired, loss of striation [114]	Strong neutrophilic infiltration after 6–12h [48]	CBN and inflammatory reactions becomes the major contributor to infarct region [68, 119]
Contraction band necrosis (CBN)	Occurrs during ischaemia and worsen during minutes after reperfusion, Reperfusion → Ca^2^⁺ overload, ROS [158]	Sarcoplasmic Ca^2^⁺ cycling, activation of calpain, MPTP opening, uncoordinated contraction [153]	Hypercontracted sarcomeres, contraction bands, ruptured membranes [190]	Condensed, banded [148]	Neutrophilic infiltration, mild to moderate [79]	Key in peri-infarct zone; contributes to infarct expansion after reperfusion [69]
Apoptosis	Intrinsic pathway via Mitochondrial ROS production and Ca^2^⁺ overload Extrinsic pathway via sarcolemmal death receptor activation [69]	Caspase-dependent; MPTP opening causes necrosis and apoptotic cell death [7, 30]	Cell shrinkage, chromatin condensation, DNA and nuclear fragmentation [69]	Membrane integrity is preserved [195]	Lack of inflammatory reaction [186]	Minor to moderate; mainly in border zones [69]
Necroptosis	Sarcolemmal TNF/Toll-like receptor activation due to low or absent of ATP [124]	RIPK1/RIPK3 → MLKL-mediated membrane pore formation [24]	Swelling, membrane rupture, no apoptotic nuclear changes	Lost [195]	Leads to inflammation due to release of cellular contents [195]	Moderate contributor; targetable by necrostatin [124]
Pyroptosis	DAMPs (e.g. mtDNA, ILs acting on sarcolemma), inflammasome activation [69]	Caspase-1 activation by inflammasomes → gasdermin D pore formation (10–20 nm diameter) [4, 92, 177]	Cell swelling, inflammasome formation, membrane pores [177]	Lost [195]	Strong, IL-1β and DAMPs released [33, 177]	Amplifies inflammation; propagates cell death to neighbour cardiomyocytes
Ferroptosis	Iron overload, glutathione → increased ROS, pathway dysfunction [193]	Inactivation of GPX4 → abnormal glutathione metabolism, lipid ROS accumulation → mitochondrial injury [69]	Damaged mitochondria, nuclear changes [34]	Iron-dependent lipid peroxidation causes membrane damage and rupture [168]	Moderate [193]	Emerging role; iron-dependent, lipid-based damage [193]
Autophagy (protective)	Stress, damaged organelles, mild ischaemia [69]	Beclin-1, Parkin, LC3 → autophagosome/lysosome fusion	Formation of double membrane, autophagosomes, organelle clearance, preserved nuclei [69]	Intact [127]	Minimal [142, 186]	Protective role via mitophagy (selective mitochondrial removal) [14]

→ lead to, *IL* interleukin, *mtDNA* mitochondrial DNA, *GPX4* Glutathione peroxidase 4, *LC3* Microtubule-associated protein light chain 3, *MLKL* mixed-lineage kinase domain-like kinase, *RIPK* receptor interacting protein kinase

In a canine model of coronary occlusion, the areas of CBN were found to be distributed predominantly to the lateral peripheries of the area at-risk [146]. This was suggested to be due to the strong force or tension on the LV wall, especially at the border zone. The cardiomyocytes situated at the boundary between the healthy and oxygen-deprived parts of the LV experience extra mechanical pressure. These myocytes face stress from two main factors. First, the dysfunctional LV segment moves in a swinging motion, pushing outward during contraction (systole) and pulling inward during relaxation (diastole). Second, there is friction on the sides between inactive oxygen-deprived cells and neighbouring active, non-damaged, beating cells [165]. Upon macroscopic examination, the infarcted zone consists of autolytic myocardium, (a phenomenon where myocardial tissue undergoes self-digestion) appearing pale due to the lack of blood supply resulting from cell injury and vessel destruction [9, 147].

In addition, gap junctions, which consist of multiple channels that allow communication between cells, appear to contribute to the development of early cardiomyocyte necrosis [135]. Garcia-Dorado et al showed that communication facilitated by this gap junction amongst ischaemic cells contributes to the spread of cellular damage during the reperfusion of the myocardium [40]. The same group later demonstrated that the spread of hypercontracture occurs via this junction between paired cardiomyocytes. These findings imply that CBN is conveyed across adjacent cells through the gap junction [154]. Subsequently, CBN is limited to the borders of the area at risk in the mid-myocardium and does not extend into the non-area at risk beyond these borders. During ischaemia, there is an observed increase in the gap junction activity, specifically in the risk zone, and this enhancement is not observed in the non-risk zone. Consequently, this selective enhancement of gap junction in the area at risk is fundamental in limiting the spread of CBN primarily to the risk zone following the reintroduction of blood flow during reperfusion [160]. The movement of Na^+^ via gap junctions from hypercontracted cells to neighbouring cells, along with the consequent alteration in cytosolic Ca^2+^ accumulation, were suggested to be the factors in the spread of contracture [154]. On the other hand, it is likely that metabolic coupling during ATP restoration in the myocardium after reperfusion leads to the formation of rigour contracture. Beyond these metabolic alterations, hypercontracted cells also interact with adjacent cells, potentially spreading the mechanical damage and cell necrosis [135].

## Therapeutic approaches and its relevance to hypercontracture

### Ischaemic conditioning

There are many cardioprotective therapies for IR that have been studied previously [54, 133]. These cardioprotection strategies can be classified according to the specific protective modality employed, the timing of their application, as well as the cellular and intracellular level they aim to target [31]. One of the extensively studied cardioprotective approaches involves the implementation of alternate short duration of ischaemia and reperfusion, referred to as ischaemic preconditioning (IPC) [52]. The cardioprotection against IR through this myocardial ‘conditioning’ is based on targeting innate cardioprotective signalling pathways [181]. The in-depth pathophysiology underlying this cardioprotection remain incompletely defined but it likely operates through multiple synergistic pathways, potentially leading to optimal cardiac protection [31, 132].

IPC is widely regarded as the most robust approach for the protection from myocardial IR injury [56, 110]. Involving several primary protein kinase cascades, the activation of receptors in cardiomyocytes contributes to the recruitment of IPC mediators. These cascades have a major impact on the propagation of the preconditioning signal [57, 58, 65]. Myocardial conditioning confers cardioprotection via protein kinase C- endothelial nitric oxide synthase-protein kinase G (PKC-eNOS-PKG), the reperfusion injury salvage kinase (RISK) pathway as well as the survivor activating factor enhancement (SAFE) pathway [59]. Wang and colleagues demonstrated the effectiveness of a preconditioning model using isolated rabbit myocytes, where simulated ischaemia offered protection against a subsequent, severe ischaemic insult. In this *in vitro* model, IPC significantly inhibits isoprenaline-induced myocyte hypercontracture (a marker of irreversible injury), and this protection involves both adenosine A1 and A3 receptor activation [184]. Our recently published data demonstrated that CBN, a hallmark of hypercontracture, was clearly distinguished by histology using PTAH staining at the onset of reperfusion in an *in vivo* model of IR injury. To evaluate the degree of CBN, an unbiased qualitative scoring system was established, showing that cardioprotective by IPC dramatically decreased the CBN score in IR rats. It in addition indicates that the tissue was protected by the IPC intervention, preventing the development of severe CBN caused by IR injury [194].

Remote ischaemic conditioning (RIC) is emerging as a more clinically relevant approach compared to conventional IPC as it safer and easier to implement in clinical settings [72]. However, to our knowledge, no studies have investigated the impact of RIC on preventing hypercontracture of myocytes specifically. Turrell et al. (2014) hypothesised that IPC offers cardioprotection by limiting Ca^2+^ accumulation during ischaemia, thereby reducing mechanical injury caused by hypercontracture via MPT pores during reperfusion. Their study compared the effects of conventional IPC and remote IPC on ventricular cardiomyocytes, examining calcium regulation and MPT pore dynamics using fluorescence microscopy under simulated IR conditions. Both forms of IPC preserved Ca^2+^ homeostasis and contractile function following the reoxygenation of metabolically inhibited cells, offering protection against IR injury. However, only conventional IPC specifically reduced Ca^2+^ loading during metabolic inhibition, independent of sarcolemmal K_ATP_ channel activity, but associated with lower sodium accumulation due to decreased Na^+^/H^+^ exchanger activity. Whilst remote IPC and conventional IPC both inhibited MPT pore opening to a similar extent, the protective mechanism in remote IPC appeared independent of Ca^2+^ loading [179].

The initiation of the RISK cascades promoting cardioprotection relies heavily on phosphoinositide 3-kinase (PI3K) [150, 151, 191]. The importance of PI3K in cardioprotection has been highlighted due to its significant role in cardiac physiology, increase of contractility, and facilitation physiological growth whilst minimising pathological hypertrophy [116]. It is well-established that constitutively active PI3K enhances the contractile function of the LV in HF. Moreover, the cardioprotective properties of insulin, an activator of PI3K, have been substantiated through experimental evidence [32, 46, 151]. Abdallah et al. used isolated cardiomyocytes to induce a hypoxia-reoxygenation model and study how insulin works in protecting cardiomyocytes from hypercontracture caused by reoxygenation. After 15 min of reoxygenation, insulin demonstrated a significant prevention of further cardiomyocytes shortening (which had already shortened during hypoxia) compared to the control group. They further showed that this inhibition of hypercontracture by insulin was abolished by the known PI3K inhibitor, LY294002, providing additional support that insulin activates its protection via the PI3K pathway. The protective effects of insulin via the PI3K pathway were linked to the activation of SERCA. They then reported that activated SERCA promotes larger cytosolic Ca^2+^ uptake by the SR. This further enhances the control of cytosolic Ca^2+^ influx upon reoxygenation, thereby preventing massive muscle contracture [1]. Yellon’s group showed that insulin could prevent hypercontracture and MPTP opening in primary cardiomyocytes subject to laser-induced ROS [32]. Subsequently, Rossello et al. performed a series of pharmacological studies to demonstrate the role of activation of the alpha isoform of PI3K preclinically. Insulin was used to promote PI3Kα activation, and this was investigated in conjunction with highly selective PI3Kα inhibitors. They demonstrated that PI3Kα isoform activity, within the first minutes of reperfusion, is crucial for minimising myocardial infarct size [151].

The effects of IPC in delaying the opening of MPTP upon reperfusion are also regarded as endogenous cardioprotective interventions in reducing cardiac injury [51, 55]. To illustrate the involvement of the RISK pathway and its effects on the MPTP, a previous study explored whether activating the pro-survival kinase pathway may provide protection to cardiomyocytes by delaying MPTP opening. MPTP opening in isolated cardiomyocytes was induced by using a fluorescent dye called tetramethyl rhodamine methyl ester (TMRM) to provoke mitochondrial reactive oxygen species (ROS) generation. As ROS production causes ATP depletion and increase Ca^2+^ accumulation [70], this MPTP opening was followed by myocyte rigour contracture. They discovered that activation of AKT expression by insulin was able to considerably delay MPTP opening, hence inhibiting myocyte contracture [32]. Whether a PI3Kα agonist can effect hypercontracture is as yet not known.

Another study introduced a novel method for protecting mitochondria, known as mitochondrial preconditioning. This technique involves pre-activating mitochondrial potassium ATP-channels (mito K_ATP_ channels) in cardiomyocytes before ischaemia [26, 123]. Preconditioning exhibits cardioprotection by inducing the opening of mitochondrial K_ATP_ channels. An earlier study by Pain et al. (2000) showed that opening of the mito K_ATP_ channels by diazoxide activates a preconditioned state. The proposed mechanism is that the opening of these channels, either during preconditioning ischaemia or diazoxide infusion, generates free radicals that initiate a memory of preconditioning. This process then activates a kinase cascade during subsequent ischaemia, ultimately modulating an effector that provides myocardial protection [126]. A later study by O’Rourke demonstrated that preconditioning reduces ischaemic damage to mitochondria and improves the restoration of cardiac function during reperfusion *ex vivo*. The opening of the mito K_ATP_ channels in this study is thought to dissipate the mitochondrial membrane potential, thereby reducing mitochondrial Ca^2+^ overload [185]. The effects of IPC were also linked to its secondary effects of reducing hypercontracture-induced mechanical injury by cytosolic Ca^2+^ from the SR. Ca^2+^ uptake and release by the SR were relatively preserved in IPC hearts following reperfusion, that might result in a more substantial SR accumulation of calcium and reduce Ca^2+^ overload-induced hypercontracture [173].

Apart from the RISK pathway, the SAFE pathway is another key mediator of cardioprotection leading to a reduction in infarct size following myocardial IR injury. This SAFE pathway comprises tumour necrosis factor α (TNFα) release and JAK-STAT3 activation [103]. Whilst the specific effector proteins inhibiting cardiomyocyte hypercontracture in this protective pathway are still debated, the SAFE pathway, activated by the immune system, induces a RISK-independent cascade involving multiple pro-survival components, including the transcription factor signal transducer and activator of transcription 3 (STAT3) that has a direct effect on maintaining mitochondrial integrity and attenuating MPTP opening [10, 37, 103]. The concept of the SAFE pathway originated from the 1999 discovery that TNFα, an immune system mediator, could confer protection against IR injury when used as a preconditioning stimulus [104, 105]. TNFα is ubiquitously expressed, including in inflammatory cells, cardiomyocytes, and fibroblasts. To delineate the cellular source of TNFα driving protective signalling, Lecour et al. utilised cardiomyocyte-specific TNFα-knockout mice. In these mice, ischaemic postconditioning neither provided protection nor activated STAT3, indicating that cardiomyocyte-derived TNF is essential for SAFE pathway-mediated cardioprotection [100].

It is also well-documented that phosphorylated STAT3 localises to both nuclear and mitochondrial compartments and play significant roles during IR injury [171, 172, 188]. Mitochondrial STAT3 requires serine phosphorylation and cyclophilin D-mediated stabilisation, plays key regulatory roles in oxidative metabolism by controlling ROS levels, ETC complexes activity, and MPTP opening [10, 11, 117]. Heusch et al. demonstrated that the molecular mechanisms underlying cardioprotective of STAT3 signalling exhibit significant differences between large mammalian species and simpler small rodent models and phosphorylation the protein residue of STAT3 within the mitochondria mediates cardioprotection by postconditioning in pig models [74, 164]. Their findings demonstrated that the infarct-sparing effect of ischaemic postconditioning was associated with elevated STAT3-tyrosine^705^ phosphorylation, and that pharmacological JAK/STAT blockade using AG490 abolished both the molecular and functional protective responses. They further showed that ischaemic postconditioning via the activation of STAT3 preserved the mitochondrial complex 1 respiration with ischaemic postconditioning and was inhibited with JAK/STAT blockade using AG490 *in vivo* and STAT3 inhibitor *in vitro* [74] Remote perconditioning study conducted by Skyschally et al. also demonstrated that enhanced STAT3 phosphorylation in the pig heart during early reperfusion (at 10 min) was directly associated with the reduction in infarct size [163].

Although the role of the SAFE pathway in delaying or inhibiting MPTP opening is well-documented as a cardioprotective mechanism following IR injury, its specific effects on modulating cardiomyocyte hypercontracture or reducing CBN along with infarct size reduction have not been well-documented previously and this highlights an open question that remains to be explored.

## Pharmacological interventions

Evidence suggests that the administration of specific cardioprotective agents or interventions during the acute ischaemic phase may reduce the occurrence of infarction. For instance, a multicentre, randomised, controlled clinical trial showed that administering intravenous (IV) (β)-blocker, metoprolol, upon reperfusion limits infarct size and enhances cardiac function in STEMI patients undergoing primary PCI [81]. The concept of studying β-blockers for cardioprotection in AMI originated more than decades ago, when propranolol was shown to delay the progression of myocardial necrosis in dogs following permanent coronary ligation [144]. The mechanism behind this β-blockade was not fully elucidated at that time, but it was suggested that it could be mediated via the inhibition of Ca^2+^ influx by SR [64]. This study provides evidence that the inhibition of contractility-induced myocardial injury by β-blockers may represent a pivotal mechanism to prevent the progression of infarction. A later study by Park et al. demonstrated that the early administration of landiolol, a short-acting β-blocker, via intracoronary injection at reperfusion, was able to exhibit protection in a pig model of IR injury. These protective effects of landiolol were associated with ultrastructural alterations, including a reduction in contraction bands within sarcomeres and swelling of mitochondria, as well as ameliorating injury to mitochondrial cristae [128]. Subsequently, IV metoprolol emerged as the β-blocker with the most extensive and well-documented testing in recent trials involving patients undergoing primary PCI [81, 149]. Although the potential long-term therapeutic benefits of metoprolol are unclear, its administration is considered safe in patients without clinical indications of HF and has demonstrated a decrease in the events of ventricular fibrillation (VF) and MVO. Subsequently, IV metoprolol is suggested to be given to STEMI patients undergoing primary PCI [23, 44, 81]. A study in a pig model was also reported to show effective treatment with metoprolol in reducing infarct size after 30 to 50 min coronary artery occlusion by intracoronary balloon, with lower no reflow as well as improved LVEF [90]. Although metoprolol may lead to α-adrenoceptor activation, which can cause vasoconstriction as a side effect, its protective effects was associated with its beneficial effect of reducing neutrophil plugging, which then improving microcirculatory flow, hence improving microcirculatory flow [43].

The role of Ca^2+^ influx in IR injury has also been widely investigated, given the central role of calcium overload in reperfusion-induced cell death. Reduction of Ca^2+^ influx by administration of NCX inhibitor, KB-R7943 was shown to prevent Ca^2+^ influx into cardiomyocytes, thereby reducing hypercontracture and cell death upon reperfusion in rat hearts [82]. Whilst some experimental studies have demonstrated protective effects of calcium blockade, clinical trials have returned mixed results. Earlier clinical studies have shown that non-dihydropyridines were associated with higher mortality rates and an increased risk of late-onset heart failure because of their negative inotropic effects. Therefore, their use should be avoided to prevent further impairment of cardiac function [29, 45]. In addition, Kim et al (2023) showed that treatment with calcium channel blockers lowers the risk of adverse cardiovascular events in AMI patients who have preserved LVEF. This protective effect of calcium channel blockers in AMI may be attributed to their ability to inhibit vasospasm. Although their findings carry significant clinical implications, several limitations must be acknowledged. Specifically, the study was based on non-randomised, observational data, and details regarding the rationale behind physicians prescribing calcium channel blockers or the dosages used were not provided. In addition, the sample size or follow-up duration may have been insufficient to fully assess the prognostic impact of calcium channel blockers therapy in AMI patients [88].

Finally, there is evidence that the infarct size could be reduced by limiting hypercontracture using cardioprotective agents in a myocardial IR injury model *in vivo*. A recent study investigated whether two, recently developed, selective cardiac myosin-targeted inhibitors could alleviate reperfusion-induced hypercontracture *in vivo*. These inhibitors, namely Mavacamten and Aficamten, were recently approved by FDA to be used in the treatment of hypertrophic cardiomyopathy amongst patients as they were shown to be well tolerated and safe [113, 166]. Since they have the ability to reduce hypercontractility by reducing actin-myosin crossbridge formation within the sarcomere, it was hypothesised that these inhibitors could be repurposed to alleviate reperfusion-induced hypercontracture *in vivo*. The inhibitors were shown to reduce infarct size when administered prior to reperfusion, without impairing haemodynamic blood pressure *in vivo* [194]. To support the *in vivo* findings, a simple model of hypercontracture *in vitro* was used, in which the inhibitors were found to significantly inhibited primary cardiomyocyte shortening and preserve cellular integrity. By mitigating hypercontracture, these inhibitors could prevent further propagation of myocardial damage during reperfusion [194]. However, their ability to reduce myocardial IR injury whilst preserving overall contractile function requires further investigation.

## Clinical implications and conclusions

Morbidity and mortality remain unacceptably high amongst STEMI patients, despite improvements having been made in intervention therapies. Whilst reperfusion initially aims to salvage myocardial cells from injury after prolonged ischaemia, it can contribute to further damage in the infarcted area. Hypercontracture may exacerbate myocardial injury and lead to arrythmias, myocardial haemorrhage and MVO. This phenomenon has important clinical implications, as it is associated with detrimental effects including as LV dysfunction and an increased risk of complications and is potentially life-threatening.

In view of the above, the major challenge in this area remains the successful translation of promising laboratory results from bench to bedside to result in positive outcomes in clinical trials. For example, predicting the extent of myocardial necrosis amongst patients with STEMI who have undergone PCI remains a significant clinical challenge. The difficulty arises because predicting the spread of necrosis in patients is not as straightforward as in animal studies, where histological observation in controlled animal experimental settings is more feasible. Histological investigations focussing on early features of myocardial injury are also rarely conducted in human heart samples these days, due to advancements in medical care and a decrease in in-hospital mortality amongst patients with AMI. Furthermore, treatments with high selectivity for hypercontracture without adverse have not yet been identified. Considering the quantity and reproducibility of robust data in animal models demonstrating the significance of hypercontracture in reperfusion injury, further clinical exploration of novel therapeutic strategies targeting hypercontracture appears justified.
